# Does Periodontal Bone Loss Play a Significant Role in Schneiderian Membrane Thickening? A Cone-Beam Computed Tomography Evaluation

**DOI:** 10.3390/medicina61091529

**Published:** 2025-08-26

**Authors:** Ebru Sarıbaş, Müzeyyen Kandemir, Mehmet Cudi Tuncer

**Affiliations:** 1 Faculty of Dentistry, Department of Periodontology, Dicle University, Diyarbakir 21090, Turkey; muzeyyenozyavuz@gmail.com; 2 Faculty of Medicine, Department of Anatomy, Dicle University, Diyarbakir 21090, Turkey; drcudi@hotmail.com

**Keywords:** cone-beam computed tomography, dental radiography, maxillary sinus, periodontal bone loss, Schneiderian membrane

## Abstract

*Background and Objectives:* Thickening of the maxillary sinus mucosa is commonly detected in asymptomatic individuals through various radiographic techniques. This study aimed to evaluate the maxillary sinus mucosal thickness (MT), also known as Schneiderian membrane thickness, in patients with periodontal bone loss (PBL) using cone-beam computed tomography (CBCT). *Material and Methods:* In this retrospective study, CBCT images from 315 patients who presented to the Department of Periodontology, Faculty of Dentistry, Dicle University, between January 2023 and January 2025 for various indications were evaluated. PBL, the presence or absence of mucosal thickening at the maxillary sinus floor, age, and gender were recorded. Statistical analyses were conducted to assess the relationships between age, gender, PBL, and MT. *Results:* A significant association was observed between PBL and MT. Sinus mucosal thickening was notably more prominent in individuals with advanced bone loss. Gender had no statistically significant effect on MT. A moderate, positive, and statistically significant correlation was found between age and MT. *Conclusions:* This study demonstrates that PBL exerts a significant and measurable impact on MT. Compared to conventional diagnostic methods, CBCT provides superior diagnostic accuracy for detecting periodontal bone defects and evaluating the soft tissue morphology at the base of the maxillary sinus.

## 1. Introduction

Periodontitis is a chronic inflammatory disease characterized by the destruction of the supporting structures of the periodontium and affects up to 90% of adults [1]. Beyond local tissue damage, periodontitis may also induce pathological changes in anatomically adjacent structures [2]. Notably, the maxillary molars located in the posterior region of the upper jaw share a close anatomical relationship with the floor of the maxillary sinus. As periodontal disease progresses, these teeth, which often have a poor prognosis, may require extraction, and dental implant therapy is frequently planned in the affected region [3]. However, the vertical bone height necessary for implant placement is often compromised by alveolar bone loss due to periodontal destruction. In such scenarios, maxillary sinus augmentation is commonly performed as a reliable and predictable surgical intervention [4].

The Schneiderian membrane lines the inner surface of the maxillary sinus and is composed of respiratory-type mucosa, including pseudostratified ciliated columnar epithelium and a highly vascularized connective tissue layer. The average physiological thickness of the membrane ranges from approximately 0.8 to 1.0 mm, rendering it difficult to detect on conventional radiographs under healthy conditions [5]. However, when the membrane undergoes inflammatory changes due to infectious or allergic stimuli, its thickness may increase up to 10–15 times the normal level, becoming radiographically visible. Periodontal infections involving posterior maxillary teeth can result in such pathological changes, including mucosal inflammation and thickening. On radiographic images, a thickened mucosa appears as a radiopaque band parallel to the bony sinus wall and lacks cortical bone characteristics [6,7].

The condition of the Schneiderian membrane plays a pivotal role in the success of both internal and external sinus floor elevation procedures. Mucosal thickening is one of the most frequently observed pathological alterations, which is reported in approximately 48% of patients and 29% of sinuses [7,8,9,10]. Significant mucosal thickening or pre-existing sinus inflammation can increase the risk of surgical complications, such as membrane perforation, hemorrhage, or exacerbation of inflammatory conditions [11]. Among these, membrane perforation is the most common complication, with reported incidence rates ranging from 8.6% to 59.8% [12].

CBCT, introduced in 1998, has emerged as a powerful three-dimensional imaging modality for evaluating the dento-maxillofacial region [13]. Compared to conventional computed tomography, CBCT provides comparable image quality at lower radiation doses, making it preferable in routine dental practice [14]. Although it has limited soft tissue contrast, CBCT is regarded as a reliable and reproducible tool for assessing sinus mucosal features, including MT [15]. Its advantages have led to its widespread use in both dental and otolaryngological fields for high-resolution imaging of the paranasal sinuses [14]. In comparison with panoramic radiography or periapical imaging, CBCT offers superior spatial resolution and eliminates the superimposition of anatomical structures, enabling more accurate visualization of the sinus membrane and surrounding bone. Furthermore, the three-dimensional capability of CBCT allows for precise measurements of MT at its maximum point, which is essential for detecting subtle changes that may not be visible on two-dimensional images. In this study, CBCT was chosen over conventional methods because it provides detailed anatomical information while maintaining a relatively low radiation dose, making it highly suitable for retrospective evaluation of both PBL and Schneiderian membrane thickness within the same dataset.

The aim of this study is to evaluate the demographic characteristics associated with and radiographic characteristics of MT and its relationship with PBL using CBCT.

## 2. Materials and Methods

### 2.1. Study Design and Ethical Approval

This retrospective observational study was conducted at the Department of Periodontology, Faculty of Dentistry, Dicle University. Ethical approval was obtained from the Dicle University Faculty of Dentistry Ethics Committee (decision No.: 2025-44). All patient data were handled in accordance with the ethical standards of the Helsinki Declaration.

### 2.2. Sample Size Calculation

The sample size calculation was based on previously published CBCT studies evaluating maxillary sinus MT in relation to PBL, which reported medium effect sizes (f ≈ 0.25) in similar populations [2,7]. These effect size estimates were used as input parameters in the G*Power 3.1 software [16] to calculate the required sample size at a 95% confidence level (α = 0.05), 80% power (1–β = 0.80), and an expected medium effect size (f = 0.25). The calculation indicated that at least 233 participants would be needed. To compensate for exclusions and ensure power, a total of 304 patients were included.

### 2.3. Patient Selection

CBCT images of 315 patients who presented to the Periodontology Clinic of Dicle University between January 2023 and January 2025 were retrospectively reviewed. Based on eligibility criteria, 304 patients were included. One sinus per patient was analyzed, chosen from the maxillary molar region with the most severe PBL. During patient selection, PBL was determined directly from CBCT images by measuring the distance from 2 mm apical to the cemento-enamel junction (CEJ) to the alveolar bone crest, as described in the Methods section. This radiographic measurement, rather than previous imaging records, was used to identify eligible cases for inclusion.

### 2.4. Inclusion and Exclusion Criteria

The inclusion criteria were age ≥ 18 years, presence of PBL in at least one posterior maxillary tooth, and availability of a complete CBCT image set. The exclusion criteria were caries, periapical lesions, root fractures, restorations, or endodontic treatment in the posterior maxilla; absence of premolar/molar teeth (excluding third molars); fluid accumulation or cystic lesions in the sinus; acute sinusitis; and presence of dental implants in the posterior region [2].

### 2.5. CBCT Software and Image Processing

All images were obtained using a three-dimensional imaging device (I-CAT^®^, Model 17–19, Imaging Sciences International, Hatfield, PA, USA). Sagittal plane alignment was guided by a laser, and the horizontal plane was set parallel to the floor and Frankfort plane. The imaging settings were as follows: voxel size: 0.3 mm; 120 kVp; 5 mA; scan time: 8–9 s. DICOM data were evaluated using i-CAT Vision software, using standardized magnification, contrast, and brightness settings for all measurements. Cross-sectional and panoramic views were used for assessment.

### 2.6. Examiner Calibration and Reliability

All radiographic measurements were performed by a single experienced periodontist. To assess intra-observer reliability, a random sample of 30 CBCT images was re-analyzed two weeks after the initial evaluation. Measurements included both MT and PBL. Intra-class correlation coefficients (ICCs) were calculated for both parameters. The ICC values were 0.93 for MT and 0.89 for PBL, indicating excellent repeatability and measurement consistency.

### 2.7. Blinding and Data Entry

To minimize observer bias, the examiner was blinded to the patient’s demographic data and clinical diagnosis during the image evaluation. All measurements were coded and entered into a pre-structured SPSS database by an independent researcher not involved in the image acquisition or interpretation.

### 2.8. Classification of Demographic Variables

The variables recorded were age, gender, MT, and PBL. Age was grouped into five categories based on the existing literature: 0–18 years (youth), 19–25 years (young adults), 26–40 years (adults), 41–60 years (middle-aged), and ≥60 years (geriatric) [17].

### 2.9. Measurement of MT

**Figure 1:**
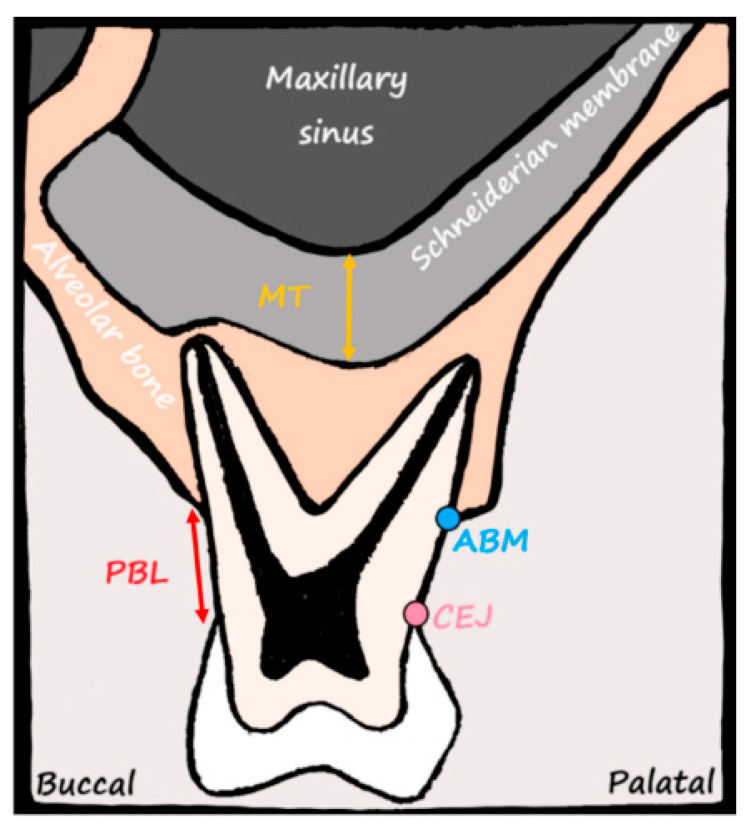
Anatomical structures of the maxillary posterior region visualized in a schematic cross-sectional view. The image illustrates the key reference points used in the CBCT-based measurements. MT is measured from the bony floor of the maxillary sinus to the outer border of the Schneiderian membrane (yellow arrow). PBL is evaluated as the distance between the CEJ and ABM (red arrow). The buccal and palatal orientations are labeled to aid spatial understanding. This figure was created by the authors specifically for this study and is not adapted from any previously published source.

MT was defined as the vertical distance from the floor of the sinus to the highest point of the thickened mucosa on the sagittal plane [8,18]. If the boundary of the sinus was indistinct and measured between 0.9 and 1 mm, a standardized value of 1 mm was used [19]. Mucosal thickening was classified into five categories [17]: Class I (≤1 mm), Class II (>1–2 mm), Class III (>2–4 mm), Class IV (>4–10 mm), and Class V (>10 mm). Figure 1 shows the anatomical reference points, including the CEJ and alveolar bone margin (ABM) in the maxillary posterior region.

**Figure 2:**
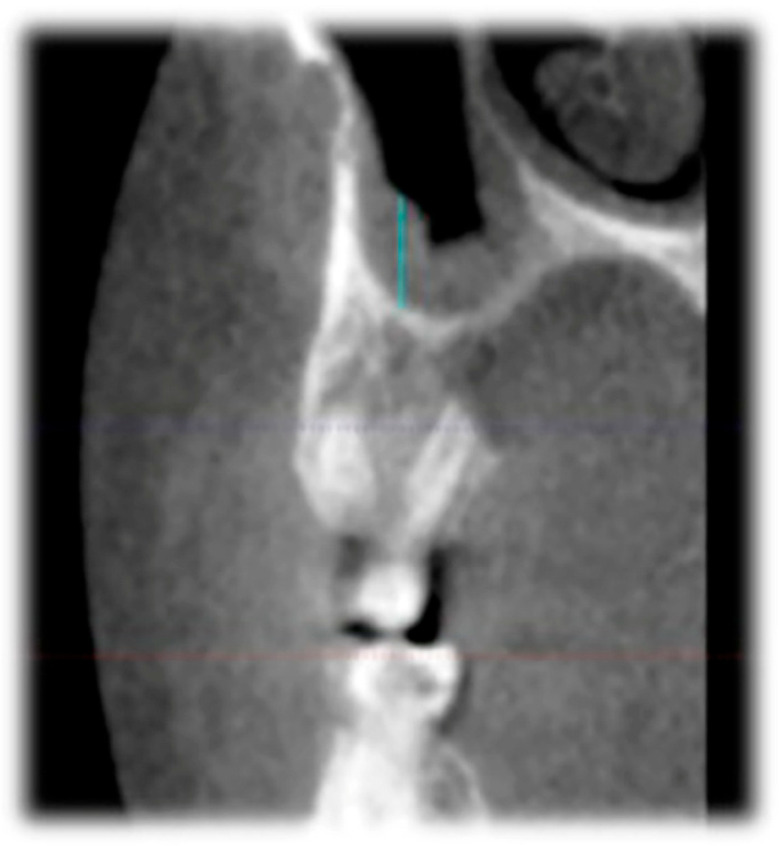
Measurement of MT on a sagittal CBCT image. The blue vertical line represents MT, measured from the bony floor of the maxillary sinus to the outermost border of the Schneiderian membrane. This value reflects the maximum MT in the evaluated region. CBCT enables precise identification of anatomical boundaries, facilitating reliable assessment of sinus membrane thickening potentially associated with periodontal or periapical pathology. In Figure 2, mucosal thickening was differentiated from mucus retention or other sinus contents by its continuous, uniform, and well-defined radiopaque appearance along the bony sinus wall. In contrast, mucus or fluid accumulation typically presents with different radiodensity patterns, lacks a consistent attachment to the sinus wall, and may exhibit air-fluid levels. Only cases with clearly demarcated mucosal thickening were included in the measurements.

In this study, CBCT was used to evaluate MT in the posterior maxillary region. On sagittal sections, the MT was measured as the perpendicular vertical distance from the most inferior point of the bony floor of the maxillary sinus to the outermost radiopaque border of the sinus mucosa. The measurement was performed at the site exhibiting the greatest mucosal thickening in the maxillary molar region. In Figure 2, the blue vertical line represents the measured MT. CBCT imaging allows for precise localization of the anatomic margins of the sinus floor and mucosa, providing high-resolution visualization suitable for assessing subtle changes in membrane thickness. All measurements were conducted using standardized contrast and magnification settings to ensure reproducibility.

### 2.10. Assessment of PBL

PBL was measured on frontal CBCT sections by calculating the distance from 2 mm apical to the CEJ to the alveolar bone crest at the mesial and distal surfaces. This measurement was then normalized by the total root length. Bone loss was classified as follows [8]: Class 0 (no bone loss), Class I (mild, <25%), Class II (moderate, 25–50%), and Class III (severe, >50%).

### 2.11. Statistical Analysis

Statistical analyses were performed using SPSS v26.0 (IBM Corp., Armonk, NY, USA). The normality of continuous variables was assessed using the Kolmogorov–Smirnov test. Non-normally distributed data are presented as the median (min–max) and mean ± SD. The Mann–Whitney U test was used for comparisons between two groups, while the Kruskal–Wallis test was applied for comparisons among more than two groups. Correlations were examined using the Spearman correlation test. Multivariate analysis was conducted via linear regression to assess the effects of age, gender, and PBL on MT. Receiver operating characteristic (ROC) curve analysis was performed to evaluate the predictive accuracy of PBL ≥ 2 mm for MT ≥ 2 mm, and cut-off values, sensitivity, specificity, and the area under the curve (AUC) are reported. A *p*-value < 0.05 was considered statistically significant.

### 2.12. Assumption Checks for Regression Analysis

Before performing the multivariate linear regression analysis, the key assumptions were assessed. The normality of residuals was evaluated using Q–Q plots and the Shapiro–Wilk test. Homoscedasticity was checked by plotting the residuals against fitted values, and multicollinearity was assessed using the Variance Inflation Factor (VIF). The assumptions were met for all parameters.

## 3. Results

### 3.1. Demographic Characteristics and General MT

A total of 304 individuals were included in the study. The mean age of the participants was 43.74 ± 11.18 years, ranging from 22 to 75 years. The average MT was 3.95 ± 1.94 mm, with a median of 3.76 mm (range: 0.47–12.4 mm). Of the participants, 51.6% were male (*n* = 157) and 48.4% were female (*n* = 147). When MT was compared between genders, the mean value was 4.14 ± 1.92 mm in females and 3.78 ± 1.95 mm in males. According to the Mann–Whitney U test, this difference was not statistically significant (*p* = 0.063) (Table 1).

### 3.2. Distribution of MT by Classification

According to the MT classification, the most prevalent group was Class IV (4–10 mm), comprising 41.12% (*n* = 125) of cases, followed closely by Class III (2–4 mm), which accounted for 40.79% (*n* = 124). Class II (1–2 mm) was observed in 12.83% (*n* = 39) of cases, while Class I (≤1 mm) was present in 4.9% (*n* = 15). Class V (>10 mm) MT was detected in only one individual (0.3%). The mean MT values for each class were as follows: 0.77 ± 0.17 mm for Class I, 1.67 ± 0.23 mm for Class II, 3.19 ± 0.57 mm for Class III, 5.73 ± 1.28 mm for Class IV, and 12.40 mm for Class V (Table 2).

### 3.3. Association Between PBL and MT

**Figure 3:**
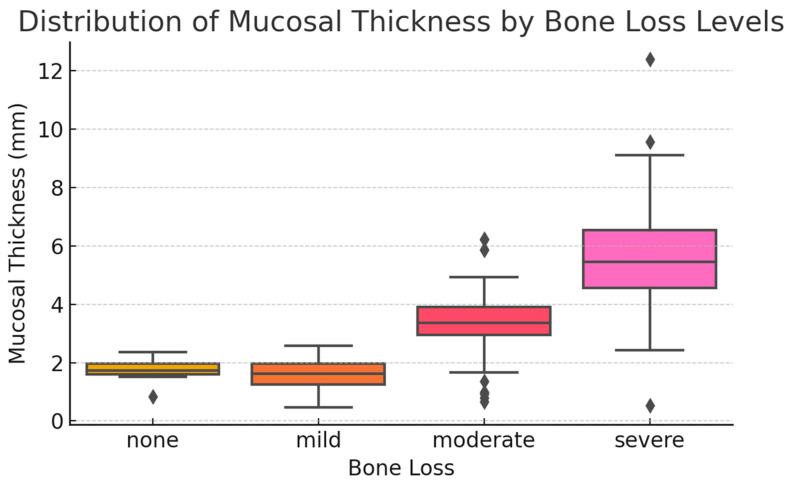
Distribution of MT according to PBL levels. Boxplot representation of MT stratified by PBL severity (none, mild, moderate, and severe). The boxes indicate the interquartile range (IQR), with the horizontal lines inside each box representing the median MT. Whiskers denote the range within 1.5× IQR, and diamonds represent outliers beyond this range. Different box colors correspond to the respective PBL categories. A clear upward trend in MT is observed with increasing severity of bone loss. Median MT values increase progressively from the “none” and “mild” categories to the “moderate” and “severe” groups, supporting a significant association between bone loss and sinus membrane thickening (*p* < 0.001, Kruskal–Wallis test).

According to the levels of PBL, 3.9% of participants (*n* = 12) had no bone loss, 13.5% (*n* = 41) had mild bone loss, 42.8% (*n* = 130) had moderate bone loss, and 39.8% (*n* = 121) had severe bone loss. When the MT values were analyzed in relation to PBL severity, the mean thickness in individuals with mild bone loss was 1.57 ± 0.56 mm, with a median of 1.62 mm (range: 0.47–2.57 mm). In those with moderate bone loss, the mean thickness was 3.36 ± 0.97 mm, with a median of 3.37 mm (range: 0.67–6.24 mm), while individuals with severe bone loss exhibited a mean thickness of 5.61 ± 1.67 mm and a median of 5.45 mm (range: 0.52–12.40 mm). For participants without bone loss, the mean MT was 1.76 ± 0.40 mm, with a median value of 1.73 mm (range: 0.83–2.37 mm). The Kruskal–Wallis test revealed a statistically significant difference in MT across the groups (*p* < 0.001) (Table 3, Figure 3).

### 3.4. Diagnostic Accuracy of Bone Loss for Predicting Mucosal Thickening

**Figure 4:**
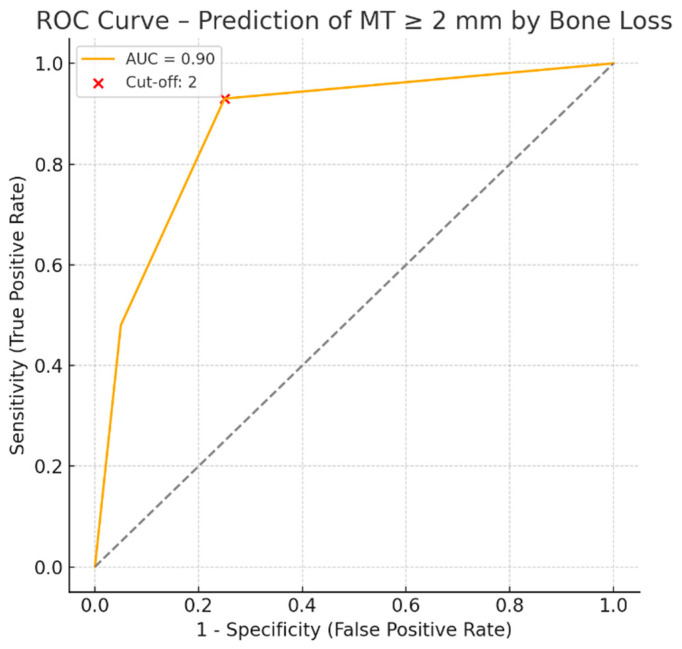
ROC curve—prediction of an MT ≥ 2 mm based on bone loss. ROC curve evaluating the predictive ability of PBL for identifying clinically relevant mucosal thickening (MT ≥ 2 mm). The optimal cut-off value for bone loss was determined to be 2 mm, with an AUC of 0.90, indicating excellent diagnostic performance. At this threshold, the model demonstrated a sensitivity of 95.2% and specificity of 75.9%. The red cross represents the optimal cut-off point on the ROC curve.

ROC analysis demonstrated that a bone loss threshold of ≥2 mm possesses significant discriminative power for predicting mucosal thickening (MT ≥ 2 mm), with an AUC of 0.90 (95% CI: 0.86–0.94, *p* < 0.001). The analysis identified 2 mm as the optimal cut-off point for bone loss in predicting clinically relevant mucosal thickening. At this threshold, the sensitivity was 95.2%, specificity was 75.9%, and the area under the ROC curve (AUC) was 0.90 (95% CI: 0.86–0.94), indicating excellent diagnostic performance. The red cross on the ROC plot represents the optimal cut-off point, determined using the Youden index (Figure 4).

### 3.5. Correlation Between Age and MT

When MT was analyzed across age groups, the mean and median values were as follows: 2.01 mm and 1.65 mm in the 19–25 age group; 3.01 mm and 3.06 mm in the 26–40 age group; 4.43 mm and 4.06 mm in the 41–60 age group; and 6.21 mm and 6.13 mm in the >60 age group. Spearman correlation analysis indicated a moderate, positive, and statistically significant correlation between age and MT (r = 0.541, *p* < 0.001) (Table 4).

### 3.6. Multiple Linear Regression Analysis

Multivariate linear regression analysis was performed to assess the effects of bone loss level, age, and gender on MT. The overall model was statistically significant (R^2^ = 0.552, *p* < 0.001). The analysis indicated that bone loss level (β = 1.548, *p* < 0.001) and age (β = 0.023, *p* = 0.009) were significant predictors of MT. In contrast, gender did not have a statistically significant effect (β = 0.190, *p* = 0.208) (Table 5).

#### Regression Assumptions

Before conducting the multivariate linear regression analysis, the key model assumptions were tested. Normality of residuals was assessed using Q–Q plots and the Shapiro–Wilk test, which indicated that the residuals followed an approximately normal distribution (Shapiro–Wilk *p* = 0.074). Homoscedasticity was confirmed through residuals versus fitted values plots, which displayed a random scatter pattern, suggesting constant variance. Multicollinearity was evaluated using VIF values, all of which were below 2, indicating no significant multicollinearity among the predictors.

## 4. Discussion

This retrospective CBCT-based study provided a comprehensive evaluation of the association between PBL and maxillary sinus MT in the posterior maxillary region. A statistically significant and progressive increase in MT was observed in parallel with increasing PBL severity, as determined by the Kruskal–Wallis test (*p* < 0.001). This correlation underscores the impact of chronic periodontal inflammation on the maxillary sinus environment. The results are consistent with previous literature suggesting that periodontal pathology may contribute to sinus membrane alterations, potentially influencing diagnostic and surgical decision-making in both dental and ENT practices.

MT in the present study was evaluated using CBCT. Due to its high-resolution three-dimensional imaging capabilities, CBCT allows for detailed assessment of anatomical structures in the maxillary sinus region. This method is considered more precise and reliable than conventional two-dimensional imaging techniques when delineating soft tissue boundaries. Brüllmann et al. reported that CBCT provides a high degree of reproducibility in measuring MT [20]. Shahbazian et al. similarly demonstrated that CBCT detects more cases of mucosal thickening than panoramic radiography, highlighting its effectiveness in identifying early-stage pathologies [21]. One study found that CBCT detected sinus membrane thickening four times more frequently than periapical radiographs, emphasizing its value in identifying the etiological origins of maxillary sinus conditions [22]. The high prevalence observed in the present study could be attributable to the detailed imaging capabilities provided by CBCT. Measurements were taken from the thickest point of the membrane, allowing for the detection of subtle thickening that might otherwise go unnoticed using less sensitive techniques. Thus, the use of CBCT in the present study enhanced the sensitivity of the findings and facilitated valid comparisons with those reported in the literature.

The literature has inconsistent findings on the effect of gender on MT. For instance, Vallo et al. reported prevalence rates of 18% for men and 8% for women [23], whereas Ren et al. found higher rates, 58.3% and 42.5%, respectively [24]. In contrast, Ramanauskaite et al. found no statistically significant difference between genders (35.1% in males vs. 28.9% in females) [12]. Consistent with the latter, no significant effect of gender on MT was observed in the current study (*p* = 0.208), suggesting that gender alone may not be a determining factor but could interact with variables such as age, periodontal status, or systemic conditions.

A moderate positive and statistically significant correlation was observed between age and maxillary sinus MT (r = 0.541, *p* < 0.001). This result is consistent with the findings of Goller-Bulut et al. who reported increased MT in individuals aged 41–60 years [8]. Phothikhun et al. similarly found that mucosal thickening is more prevalent among males and individuals aged over 49 years [7]. The trend is further supported by Lu et al. [9], although Rege et al. reported greater thickening in males but found no correlation with age [25]. Such inconsistencies may reflect differences in study populations, age ranges, sample sizes, or CBCT protocols.

The mean MT was 3.95 ± 1.94 mm, consistent with previous reports [8,26]. In the literature, the threshold for pathological thickening varies; an MT of approximately 1 mm is generally regarded as normal [7,8,10], whereas values above 2 mm are often classified as pathological [26,27,28]. The ≥2 mm criterion for maxillary sinus MT was met in 82.2% of the 304 cases. This is higher than the prevalence rates reported by Pazera et al. (46.8%) and Ritter et al. (56.3%) [29,30], but comparable to Brüllmann et al.’s 74% [20]. The high prevalence observed may be attributed to the predominance of individuals with PBL, a condition that can increase susceptibility to sinus inflammation. Periodontal destruction in close proximity to the sinus floor may facilitate the diffusion of inflammatory mediators into the Schneiderian membrane, leading to thickening.

The multivariate regression analysis identified bone loss level as the strongest predictor of mucosal thickening. Furthermore, the ROC analysis showed that bone loss was highly accurate in predicting an MT ≥ 2 mm. Zhang et al. also found a significant association between advanced bone loss and MT in a CBCT study of over 400 individuals [2]. Phothikhun et al. reported a 66.8% prevalence of MT and emphasized its close association with bone loss [7]. Similarly, Sheikhi et al. observed that individuals with poor periodontal health had average mucosal thicknesses exceeding 3 mm [18].

The biological basis for this relationship may involve inflammatory cytokines associated with periodontitis. Elevated levels of interleukin-1β (IL-1β), tumor necrosis factor-alpha (TNF-α), and interleukin-6 (IL-6) promote tissue destruction and bone resorption. IL-1β enhances osteoclast activity and upregulates matrix metalloproteinases, contributing to bone breakdown [31]. TNF-α increases inflammation by recruiting neutrophils and promoting vascular permeability, while IL-6 supports chronic inflammation via B-cell activation [32,33]. These cytokines may infiltrate the maxillary sinus via the thin alveolar bone, especially in advanced bone loss cases, resulting in Schneiderian membrane edema, thickening, and chronic inflammation [34]. Histopathological findings in odontogenic sinusitis frequently reveal epithelial hyperplasia, goblet cell proliferation, and subepithelial lymphocytic infiltration—consistent with cytokine-mediated remodeling.

The proximity of periodontal lesions to the sinus floor facilitates the transmission of inflammation to the Schneiderian membrane, leading to thickening and mucocele-like changes. Advanced bone loss further reduces the barrier between inflamed periodontal tissues and the sinus, exacerbating mucosal alterations. The ROC analysis revealed that bone loss ≥ 2 mm could predict an MT ≥ 2 mm with 95.2% sensitivity and 75.9% specificity (AUC = 0.90). This indicates a strong association between pathological mucosal thickening and periodontal status. Therefore, comprehensive periodontal evaluation should be integral to the preoperative assessment for sinus surgeries. These findings are consistent with those reported by Guo et al., Phothikhun et al., Sheikhi et al., and Zhang et al. [4,7,18,26].

Significant thickening of the Schneiderian membrane may increase the risk of complications during sinus augmentation and implant placement. Thickened membranes are more prone to perforation, hemorrhage, and impaired graft integration [11]. Membrane perforation rates as high as 59% have been reported in cases with pre-existing sinus pathology [35]. Chronic mucosal thickening may also predispose patients to postoperative sinusitis and reduce the success of sinus lift procedures. For this reason, thorough radiographic assessment of the sinus membrane is essential during preoperative planning, and any pathological thickening should be addressed before surgery.

This study has several limitations. First, the primary objective was to assess the relationship between radiographically measured PBL and Schneiderian membrane thickness, independent of detailed periodontal status parameters such as probing depth or clinical attachment loss. As this was a retrospective radiographic analysis, clinical periodontal records were not consistently available for all patients and therefore were not incorporated into the analysis. Second, due to its retrospective and cross-sectional design, only associations and not causation can be inferred. Additionally, because the participants were recruited from a single center within a specific geographic region, the generalizability of the results may be limited. Systemic and behavioral factors such as smoking and diabetes, which are known to influence both periodontal and sinus health, were not accounted for. Smoking may impair mucociliary function and promote subclinical inflammation [25], while diabetes can disrupt immune responses and exacerbate tissue inflammation [36]. Furthermore, CBCT imaging has inherent limitations, including relatively low soft tissue contrast compared with conventional CT, susceptibility to artifacts from metallic restorations, and inability to directly assess histopathological changes in the Schneiderian membrane. These limitations should be considered when interpreting mucosal measurements obtained through CBCT. Future studies should systematically address these confounding factors to better isolate the specific effects of PBL on Schneiderian membrane changes.

Future research should focus on prospective, multicenter studies with larger and more diverse populations to enhance the generalizability of the findings and confirm the relationship between PBL and MT. Longitudinal designs would be particularly valuable for clarifying the temporal sequence and potential causality between chronic periodontal inflammation and sinus mucosal alterations. Moreover, integrating systemic health parameters such as smoking status, glycemic control, and immunological profiles could help delineate the influence of confounding factors on sinus membrane morphology. Histopathological correlations with radiological findings could provide further insight into the inflammatory and structural changes occurring in the Schneiderian membrane. Additionally, evaluating the effect of periodontal treatment on reducing mucosal thickening may offer practical guidance for optimizing pre-surgical planning in sinus augmentation and implantology. Advances in imaging technologies and artificial intelligence-assisted CBCT analysis may also contribute to more accurate and automated detection of early mucosal changes, improving diagnostic efficiency and surgical outcomes.

## 5. Conclusions

The present study demonstrates a clear and progressive association between PBL and MT in the posterior maxillary region. As bone loss increased, the MT also became more pronounced, with advanced cases frequently exhibiting pathological membrane dimensions (≥2 mm). Age was identified as an independent predictor of MT, while gender showed no significant effect. The high predictive accuracy observed in the ROC analysis underscores the diagnostic value of assessing bone loss when anticipating sinus membrane alterations. These findings highlight the importance of incorporating comprehensive periodontal evaluation into the preoperative planning of maxillary sinus interventions. Adopting a multidisciplinary approach that integrates periodontology, radiology, and surgical expertise may help reduce surgical complications and improve patient outcomes. Further research should explore whether periodontal therapy can reverse mucosal changes and clarify the inflammatory pathways underlying the relationship between PBL and sinus membrane pathology.

## Figures and Tables

**Table 1:** Comparison of MT values by gender.

Gender	*n* (%)	Mean ± SD (mm)	Median (Min–Max) (mm)	*p*-Value ^1^
Male	157 (51.6)	3.77 ± 1.94	3.65 (0.65–12.40)	
Female	147 (48.4)	4.13 ± 1.91	3.91 (0.47–9.56)	0.063

**^1^ Statistical test:** Mann–Whitney U test. **Note**: Data are presented as mean ± standard deviation, median (minimum–maximum), and *n* (%).

**Table 2:** Distribution of MT by class.

MT	*n*	%	Mean ± SD	Median (Min–Max)
Class I (≤1 mm)	15	4.93	0.77 ± 0.17	0.79 (0.47–0.98)
Class II (1–2 mm)	39	12.83	1.67 ± 0.23	1.73 (1.12–1.98)
Class III (2–4 mm)	124	40.79	3.19 ± 0.57	3.26 (2.01–3.98)
Class IV (4–10 mm)	125	41.12	5.73 ± 1.28	5.54 (4.01–9.56)
Class V (>10 mm)	1	0.33	12.40	12.40

Note: Descriptive data are presented as mean ± standard deviation, median (minimum–maximum), and *n* (%). No statistical comparison was performed across thickness classes as this is a descriptive categorization.

**Table 3:** Comparison of MT according to PBL levels. This table presents the distribution of MT across the different levels of PBL. A total of 304 individuals were categorized into four groups: no bone loss and mild, moderate, and severe bone loss. The highest mean MT was observed in the severe bone loss group (5.61 ± 1.67 mm), followed by the moderate (3.36 ± 0.97 mm), none (1.76 ± 0.40 mm), and mild groups (1.57 ± 0.56 mm). The median MT values followed a similar trend. Statistical comparison using the Kruskal–Wallis test revealed a highly significant difference among the groups (*p* = 0.000197), indicating a strong association between increased bone loss and increased MT. The data are presented as the mean ± standard deviation, median (minimum–maximum), and *n* (%). * *p* < 0.001 indicates high statistical significance (Kruskal–Wallis test).

Bone Loss	*n*	%	Mean ± SD	Median (Min–Max)
None	12	3.9	1.76 ± 0.40	1.73 (0.65–2.57)
Mild	41	13.5	1.57 ± 0.56	1.62 (0.47–2.57)
Moderate	130	42.8	3.36 ± 0.97	3.37 (0.67–6.24)
Severe	121	39.8	5.61 ± 1.67	5.45 (0.52–12.40)
			*p* = 0.000197 *	

Statistical test: Kruskal–Wallis test. Note: Data are presented as mean ± standard deviation, median (minimum–maximum), and *n* (%). Kruskal–Wallis test was used for group comparison. * *p* < 0.001 was considered statistically significant.

**Table 4:** MT values according to age groups.

Age Group (Years)	*n*	Mean ± SD (mm)	Median (Min–Max) (mm)
19–25	11	2.01	1.65
26–40	112	3.01	3.06
41–60	159	4.43	4.06
>60	22	6.21	6.13

Note: Data are presented as mean ± standard deviation and median (minimum–maximum). A positive correlation between age and MT was observed (r = 0.541, *p* < 0.001).

**Table 5:** Multivariate linear regression analysis of factors associated with MT. This table summarizes the regression coefficients (β), standard errors, t-values, and *p*-values for each variable included in the model. The analysis indicates that both PBL and age were significantly associated with increased MT (*p* < 0.001 and *p* = 0.009, respectively). Gender did not have a statistically significant effect (*p* = 0.208). The model had a good explanatory power (R^2^ = 0.552).

Variable	Coefficient (β)	Standard Error	t-Value	*p*-Value
Bone Loss	1.5483	0.1199	12.9162	<0.001
Age	0.0228	0.0087	2.6296	0.009
Gender	0.1895	0.1501	1.2627	0.208

Note: Model explanatory power: R^2^ = 0.552.

## Data Availability

The original contributions presented in this study are included in the article. Further inquiries can be directed to the corresponding author.
